# Supplementary material for: Content-rich biological network constructed by mining PubMed abstracts
Source: BMC Bioinformatics. 2004 Oct 8;5:147. doi: 10.1186/1471-2105-5-147 (PMC528731; doi:10.1186/1471-2105-5-147)
Supplement: Additional File 5 — The original Chilibot query results of the term "long-term potentiation (LTP)" and 22 other terms, limiting the latest references analyzed to the years 1990, 1995, 2000, and 2004. [file 1471-2105-5-147-S5.bz2 › chilibotAdditionalFile5/ltp2000/html/ERK_PI-3K.html]

 


 **ERK** and **PI-3K** 
  
Found 126 abstracts in PubMed,  **30 abstracts were retrieved and analyzed**.  


---

 Search Google  |
 PDF files only 
|  EDU domain only 

---

**Interactive relationship** (e.g. stimulation, inhibition, etc)

- We have previously shown that inhibition of phosphatidylinositol PI 3 kinase  [ **PI-3K** ]  severely attenuates the activation of extracellular signal regulated kinase  **Erk**  following engagement of integrin fibronectin receptors.  Ref: 10801448 Curr Biol, 2000
- Inhibition by wortmannin of PI3 kinase  [ **PI-3K** ]  activity, which has been implicated in ET 1 and other G protein coupled receptor mediated signaling pathways, reduced  **Erk**  2 activation by ET 1 but had no effect on ET 1 induced EGF R and Shc phosphorylation.  Ref: 11016663 Cancer Res, 2000
- Unexpectedly, a CSF 1R lacking the PI3 kinase  [ **PI-3K** ]  binding site DeltaKI remained capable of activating MEK  **ERK**  in a PI3 kinase  [ **PI-3K** ]  dependent manner.  Ref: 10958675 Mol Cell Biol, 2000
- In the myeloid progenitor 32D cell line expressing CSF 1 receptor CSF 1R, CSF 1 activation of the extracellular signal regulated kinase  **ERK**  pathway is both Ras and phosphatidylinositol 3 kinase PI3 kinase  [ **PI-3K** ]  dependent.  Ref: 10958675 Mol Cell Biol, 2000
- These results suggest that functional responses may be dependent on the activity of PI3 kinase  [ **PI-3K** ] , which regulates at least 2 important signaling pathways by regulating activation of ras for the MEK  **ERK**  pathway and the increase in Ca i.  Ref: 10979966 Blood, 2000
- In perfused rat liver, PI 3 kinase  [ **PI-3K** ]  inhibitors largely abolished the stimulatory effect of TUDC on taurocholate excretion, suggesting an important role for a PI 3 kinase  [ **PI-3K** ]  Ras  **Erk**  pathway in the choleretic effect of TUDC.  Ref: 10926845 Biochem J, 2000
- LXA 4 activation of  **erk**  was insensitive to PTX and PI 3 kinase  [ **PI-3K** ]  inhibition.  Ref: 10869343 J Biol Chem, 2000
- PI3 kinase  [ **PI-3K** ]  inhibitors enhanced CSF 1 stimulated A Raf and c Raf 1 activities, and dominant negative A Raf but not dominant negative c Raf 1 reduced CSF 1 provoked  **ERK**  activation, suggesting that A Raf mediates a part of the stimulatory signal from Ras to MEK  **ERK** , acting in parallel to PI3 kinase  [ **PI-3K** ] .  Ref: 10958675 Mol Cell Biol, 2000
- Together, these data suggest that in airway smooth muscle ASM cells, PI 3 kinase  [ **PI-3K** ]  regulates transcription from the cyclin D 1 promoter and DNA synthesis in an  **ERK**  independent manner.  Ref: 11017907 Am J Respir Cell Mol Biol, 2000

**Parallel relationship** (e.g. studied together, co-existance, homology, etc.)

- These data suggest that VEGF can increase CTGF gene expression in bovine retinal capillary cells via KDR or Flt receptors and the activation of PI3 kinase  [ **PI-3K** ]  Akt pathway independently of PKC or Ras  **ERK**  pathway, possibly inducing the fibrosis observed in retinal neovascular diseases.  Ref: 11018037 J Biol Chem, 2000
- Muscarinic stimulation of intact muscle strips 10 microM ACh activated PI 3 kinase  [ **PI-3K** ]  gamma,  **ERK**  and p38 MAP kinases, and MAP kinase activated protein kinase 2.  Ref: 10913001 Am J Physiol Cell Physiol, 2000
- 3 PI3 kinase  [ **PI-3K** ]  affected the 12 S HETE stimulated Raf MEK  **ERK**  cascade at the level of MEK.  Ref: 10964724 Biochem Biophys Res Commun, 2000
- Muscarinic receptor activation also stimulated the mitogen activated protein kinase extracellular signaling regulated kinase MAPK  **ERK**  and phosphoinositide PI 3 kinase  [ **PI-3K** ]  signaling pathways.  Ref: 11042677 Cell Death Differ, 2000
- We assessed the role of the  **ERK**  and PI3 kinase  [ **PI-3K** ]  pathways in neonatal H I brain injury in the presence and absence of BDNF.  Ref: 10908618 J Neurosci, 2000
- Extracellular signal regulated kinase  **erk**  1 and 2 proteins and PI 3 kinase  [ **PI-3K** ]  were activated in embryonic corneal epithelia after 1 hour stimulation by type I collagen.  Ref: 11006227 Invest Ophthalmol Vis Sci, 2000
- We have investigated the role of the extracellular signal regulated kinase  **ERK** , p38 and phosphatidylinositol 3 kinase PI 3 kinase  [ **PI-3K** ]  pathways in the regulation of intestinal fructose transport.  Ref: 10926840 Biochem J, 2000
- **Erk**  and PI 3 kinase  [ **PI-3K** ]  are necessary for collagen binding and actin reorganization in corneal epithelia.  Ref: 11006227 Invest Ophthalmol Vis Sci, 2000
- The results therefore suggested that the muscarinic survival effect is mediated by a pathway that leads to caspase inhibition by MAPK  **ERK**  and PI 3 kinase  [ **PI-3K** ]  independent signaling cascades.  Ref: 11042677 Cell Death Differ, 2000
- It is concluded that there is extensive cross talk between the  **ERK** , p38 and PI 3 kinase  [ **PI-3K** ]  pathways in their control of brush border fructose transport by modulation of both the levels and intrinsic activities of GLUT5 and GLUT2.  Ref: 10926840 Biochem J, 2000
- Using coimmunoprecipitation analysis, the role of PYK2 as an upstream regulator of both extracellular signal related kinase  **ERK**  1 2 mitogen activated protein kinase and phosphatidylinositol 3 kinase PI 3 kinase  [ **PI-3K** ]  pathways was examined in cultured rat aortic VSMC.  Ref: 11121380 Am J Physiol Cell Physiol, 2001
- LTD 4 induced mesangial cell proliferation required both extracellular related signal regulated kinase  **erk**  and PI 3 kinase  [ **PI-3K** ]  activation and may involve platelet derived growth factor receptor transactivation.  Ref: 10869343 J Biol Chem, 2000
- Rather, transformation by Notch requires active signals from the  **Erk**  MAP kinase and PI 3 kinase  [ **PI-3K** ]  pathways downstream of Ras.  Ref: 10980592 Oncogene, 2000
- Our findings suggest that cisplatin induced DNA damage causes the phosphorylation of both BAD Ser 112 via an extracellular signal regulated protein kinase  **ERK**  cascade and BAD Ser 136 via a PI 3K  [ **PI-3K** ]  protein kinase B Akt cascade.  Ref: 11085518 Cancer Res, 2000
- Pharmacological inhibition of  **ERK** ... inhibited the ability of BDNF to block H I induced caspase 3 activation and tissue loss.  Ref: 10908618 J Neurosci, 2000
- Wortmannin 25 microM abolished the activation of PI 3 kinase  [ **PI-3K** ]  gamma,  **ERK** , and p38 MAP kinases.  Ref: 10913001 Am J Physiol Cell Physiol, 2000
- The upregulation of PLCbeta3 by IGF I is dependent on the activity of tyrosine kinase,  **ERK** , PI3 kinase  [ **PI-3K** ] , and p70 S6 kinase and PLCbeta3 expression seems to be required for the induction of immediate early genes by IGF I.  Ref: 10944430 Biochem Biophys Res Commun, 2000
- Inhibition of the PI 3 kinase  [ **PI-3K** ]  and AKT protein kinase B...  **ERK** , or JNK signaling pathways, reduced ILK induction of cyclin D1 expression.  Ref: 10915780 J Biol Chem, 2000
- Inhibition of PI3 kinase  [ **PI-3K** ]  by selective inhibitor LY294002 abolished anti IgE antibody but not FMLP induced phosphorylation of MEK1 MAPK kinase  **ERK**  kinase and ERKs while inhibiting LTC4 generation as well as histamine release.  Ref: 10979966 Blood, 2000
- LTD 4 stimulated the MAP kinases  **erk**  and p38 via a pertussis toxin PTX sensitive pathway dependent on PI 3 kinase  [ **PI-3K** ]  and protein kinase C activation.  Ref: 10869343 J Biol Chem, 2000
- The upregulation of PLCbeta3 by IGF I was abolished by preincubation of cardiomyocytes with the IGF I receptor antagonist IGF I analogue, the tyrosine kinase inhibitor genistein, the extracellular signal related kinase  **ERK**  inhibitor PD 98059, the phosphatidylinositol 3 PI 3 kinase  [ **PI-3K** ]  inhibitor wortmannin and the p70 S6 kinase inhibitor rapamycin.  Ref: 10944430 Biochem Biophys Res Commun, 2000
- The tissues were evaluated for collagen stimulated actin reorganization,  **erk**  1 and 2 and PI 3 kinase  [ **PI-3K** ]  activity, total filamentous actin accumulation, and collagen binding.  Ref: 11006227 Invest Ophthalmol Vis Sci, 2000
- Activation of extracellular signal regulated kinase  **ERK** , the archetypal mitogen activated protein MAP kinase, and phosphatidylinositol 3 PI3 kinase  [ **PI-3K** ]  are critical steps in Ang II induced mitogenic signaling.  Ref: 10916078 Kidney Int, 2000
- In addition, the same nanomolar concentrations of AG 1478 that were effective in EGF signaling blocked the Ang II induced activation of  **ERK**  and PI3 kinase  [ **PI-3K** ]  in a dose dependent manner.  Ref: 10916078 Kidney Int, 2000
- PI 3 kinase  [ **PI-3K** ]  and  **erk**  1 and 2 signaling pathways are activated and necessary for collagen binding and integrin mediated actin reorganization in embryonic avian corneal epithelium.  Ref: 11006227 Invest Ophthalmol Vis Sci, 2000
- Using wortmannin and LY 294002 to inhibit PI 3 kinase  [ **PI-3K** ]  activity and PD 098059 and SB 203580 to inhibit  **ERK**  and p38 MAP kinases, we established that these enzymes are functionally important for regulation of chemotactic migration of colonic myocytes.  Ref: 10913001 Am J Physiol Cell Physiol, 2000
- The PI 3 kinase  [ **PI-3K** ]  pathway inhibitors wortmannin and LY294002, as well as the MAPK  **ERK**  pathway PD98059 inhibitor, did not however suppress the inhibitory effect of the muscarinic receptors on caspase activity.  Ref: 11042677 Cell Death Differ, 2000
- In cultured muscle cells, newborn calf serum 3% activated PI 3 kinase  [ **PI-3K** ]  alpha and PI 3 kinase  [ **PI-3K** ]  gamma isoforms,  **ERK**  and p38 MAP kinases, and stimulated chemotactic cell migration.  Ref: 10913001 Am J Physiol Cell Physiol, 2000
- On the other hand, rottlerin the selective PKCdelta inhibitor, SB 203580 the selective p38 MAPK inhibitor, and wortmannin the PI 3 kinase  [ **PI-3K** ]  inhibitor can affect neither cPLA 2 nor  **ERK**  phosphorylation.  Ref: 10996851 J Cell Biochem, 2000
